# Functional studies of *E*. *faecalis* RNase J2 and its role in virulence and fitness

**DOI:** 10.1371/journal.pone.0175212

**Published:** 2017-04-06

**Authors:** Peng Gao, Kenneth L. Pinkston, Agathe Bourgogne, Barbara E. Murray, Ambro van Hoof, Barrett R. Harvey

**Affiliations:** 1 Center for Molecular Imaging, Brown Foundation Institute of Molecular Medicine for the Prevention of Human Diseases, The University of Texas Health Science Center at Houston, Houston, Texas, United States; 2 Division of Infectious Diseases, Department of Internal Medicine, The University of Texas Health Science Center at Houston, Houston, Texas, United States; 3 Department of Microbiology and Molecular Genetics, The University of Texas Health Science Center at Houston, Houston, Texas, United States; University of Florida, UNITED STATES

## Abstract

Post-transcriptional control provides bacterial pathogens a method by which they can rapidly adapt to environmental change. Dual exo- and endonucleolytic activities of RNase J enzymes contribute to Gram-positive RNA processing and decay. First discovered in *Bacillus subtilis*, RNase J1 plays a key role in mRNA maturation and degradation, while the function of the paralogue RNase J2 is largely unknown. Previously, we discovered that deletion of the *Enterococcus faecalis rnjB* gene significantly attenuates expression of a major virulence factor involved in enterococcal pathogenesis, the Ebp pili. In this work, we demonstrate that *E*. *faecalis rnjB* encodes an active RNase J2, and that the ribonuclease activity of RNase J2 is required for regulation of Ebp pili. To further investigate how *rnjB* affects *E*. *faecalis* gene expression on a global scale, we compared transcriptomes of the *E*. *faecalis* strain OG1RF with its isogenic *rnjB* deletion mutant (Δ*rnjB*). In addition to Ebp pili regulation, previously demonstrated to have a profound effect on the ability of *E*. *faecalis* to form biofilm or establish infection, we identified that *rnjB* regulates the expression of several other genes involved in bacterial virulence and fitness, including *gls24* (a virulence factor important in stress response). We further demonstrated that the *E*. *faecalis* RNase J2 deletion mutant is more sensitive to bile salt and greatly attenuated in *in vivo* organ infection as determined by an IV-sublethal challenge infection mouse model, indicating that *E*. *faecalis* RNase J2 plays an important role in *E*. *faecalis* virulence.

## Introduction

Overwhelming evidence supports the importance of RNA metabolism, including RNA degradation and processing, in post-transcriptional gene regulation processes in many bacterial species [1,2]. In the Gram-negative bacterium *Escherichia coli*, it is well studied that endo- and exonucleases, including three main rate-determining enzymes RNase E, RppH, and PcnB, are involved in RNA degradation and processing [3–5]. In comparison, the enzymes that regulate RNA degradation and processing in Gram-positive bacteria have been studied largely in *Bacillus subtilis* [6]. While orthologs of the enzymes for *E*. *coli* RNA metabolism are absent in *B*. *subtilis*, members of the RNase J family, together with RNase Y and RNase III, are key enzymes in *B*. *subtilis* RNA metabolism [7,8]. Two distinct RNase Js, J1 and J2, have been identified in *B*. *subtilis* which possess unique dual activity functioning as both endoribonucleases and 5’ to 3’ exoribonucleases, with the later activity preferring substrates with 5’ monophosphates over 5’ triphosphates [7,8]. *B*. *subtilis* RNase J1 is an essential enzyme and believed to be involved in many biological processes including global mRNA degradation, maturation of 16S rRNA [7] and scRNA [9], regulation of the *trp* operon and regulation of *thrS* gene expression via cleavage of leader sequences. In contrast, the function of *B*. *subtilis* RNase J2 is largely unknown. In fact, deletion of *B*. *subtilis* RNase J2 does not have a known growth phenotype [10], and no endogenous substrates for RNase J2 have been thoroughly characterized. Microarray analysis of an RNase J2 deletion mutant in *B*. *subtilis* failed to identify mRNAs that were significantly regulated by RNase J2 [10]. In addition, it was demonstrated that *B*. *subtilis* RNase J2 has significantly lower exonuclease activity compared to J1, although the endonuclease activities are similar for these two enzymes. Due to lack of substrates and reduced endonuclease activity, it was speculated that RNase J2 is functionally redundant to J1 and acts to substitute or regulate J1 [11].

Enterococci are a versatile group of bacteria found in various habitats including the gastrointestinal track of humans and animals, soil, water and food supply. Although they have been used as probiotic bacteria for animals and humans for decades, enterococci are also among the leading causes of nosocomial infections including urinary tract infections, endocarditis and bacteremia. Treatments for enterococcal infection often fail due to this organism’s ability to acquire antibiotic resistance and adapt to harsh environments. Some mechanisms that regulate *E*. *faecalis* virulence factor transcription have been characterized [12,13], but several recent studies have suggested that post-transcriptional regulation at the RNA level through riboswitches and small regulatory RNA (sRNA) also play an important role in enterococcal gene regulation and pathogenesis. One example is that up-regulation of the ethanolamine utilization genes, *eut* genes, occurs at the post-transcription initiation level and is mediated by a small regulatory RNA (eutX) that contains an AboCbl-binding riboswitch [14–16] in *E*. *faecalis* and *Listeria monocytogenes*. Additional small regulatory RNAs have been identified as important regulatory elements in *E*. *faecalis* gene expression. [17]. However, the involvement of RNA metabolism in *E*. *faecalis* post-transcriptional regulation is largely unknown. Blast search of the *E*. *faecalis* genome revealed that *E*. *faecalis* have clear orthologs of *B*. *subtilis* RNase J1 and J2 *(ef2924 for B*. *subtilis* RNase J1 and *ef1185* for *B*. *subtilis* RNase J2 with 70% and 47% sequence identity, respectively). In contrast, we have observed that most of the enzymes that are important for initiating RNA decay in *E*. *coli* are missing from the *E*. *faecalis* genome (i.e. RNase E, RppH, PcnB) [18,19].Our laboratory previously reported that the *E*. *faecalis ef1185 (rnjB)* gene, which encodes a putative RNase J2, regulates surface display of *E*. *faecalis* endocarditis and biofilm-associated pilus (Ebp), a major contributor to *E*. *faecalis* virulence [20]. The *E*. *faecalis* RNase J2 deletion mutant (Δ*rnjB)* has a clear phenotype of decreased Ebp pilus production, making *E*. *faecalis* an ideal organism in which to study the role of RNase J2 in gene expression. The finding that *E*. *faecalis rnjB* gene is involved in Ebp regulation provided motivation for further investigation of the involvement of RNases and RNA processing in *E*. *faecalis*.

In this manuscript, we characterize the gene product of *E*. *faecalis rnjB* as an active RNase J2 enzyme. The association between RNase J2 ribonuclease activity and Ebp pilus expression is determined. Further, to obtain a global view of the role of RNase J2 in *E*. *faecalis* gene expression, we performed DNA microarray analysis to identify additional genes of interest regulated by RNase J2, which were subsequently confirmed by analyzing individual gene products at the RNA and protein level. Finally, we evaluated the effect of an *rnjB* deletion in *E*. *faecalis* fitness by monitoring stress response and virulence of the mutant in a bile salt resistance assay and a mouse sublethal challenge model respectively.

## Materials and methods

### Bacterial strains, media, chemicals and primers

The strains used in this study were OG1RF, its isogenic deletion mutant *ΔrnjB*[20], and insertion mutant TX10100 (*gls24* disruption) [21]. Brain heart infusion (BHI) broth and tryptic soy broth without glucose (TSB) were purchased from Difco Laboratories (Detroit, MI). All chemicals were purchased from Sigma (St. Louis, MO). Oligonucleotides used in this study were purchased from Sigma (The Woodlands, TX) and are listed in Table 1.

**Table 1:** Primers used in this study

RNase J1 Primers for cloning:
2924BamF: 5’-GCGCGGATCCATGAAAGTAAACATAAAAAATAACG-3’
2924KpnR: 5’-CCGGGGTACCTTATTATTGATCACTAACTGTGG-3’
RNase J2 Primers used for cloning:
1185NdeF: 5’-GCGCCATATGAGTACAATAAAAATCG-3’
1185XhoR: 5’-CCGGCTCGAGCTATGCGTTATTTTTGG-3’
RNase J2 mutagenesis primers:
H69AFor: 5’- GGGGTCTTTTTAACAGCTGGCCATGCTGATGC-3’
H69ARev: 5’- GCATCAGCATGGCCAGCTGTTAAAAAGACCCC-3’
H71AFor: 5’- GGTCTTTTTAACACATGGCGCTGCTGATGCAATTGGGG-3’
H71ARev: 5’- CCCCAATTGCATCAGCAGCGCCATGTGTTAAAAAGACC-3’
D73AFor: 5’- CACATGGCCATGCTGCTGCAATTGGGGCCTTACC-3’
D73ARev: 5’- GGTAAGGCCCCAATTGCAGCAGCATGGCCATGTG-3’
qRT-PCR primers:
pyrRqRT-For: AACGAGCGCTTACTCGAATCTCGT
pyrRqRT-Rev: TTAAGCGTTCGGCTAGACGTTGTG
23SqRT-For: GTGATGGCGTGCCTTTTGTA
23SqRT-Rev: CGCCCTATTCAGACTCGCTTT

### Immuno-electron microscopy

Enterococci were grown in BHI broth, washed in 0.1 M NaCl solution and resuspended in PBS. Monoclonal antibody mAb 9 against EbpA was developed in house using recombinant EbpA protein as immunogen [22] following standard hybridoma generation methods previously described [20]. Immunogold labeling was performed using mAb 69 against EbpC [20], mAb 9 against EbpA, or mAb70 against Ace[23] for primary binding, followed by gold bead-conjugated donkey anti-mouse IgG, using methods previously described [24]. Samples were viewed on a Jeol 1400 transmission electron microscope.

### Cloning and mutagenesis

The 1689 bp DNA fragment containing the *E*. *faecalis rnjB* gene was PCR amplified from the OG1RF genome using primer pair 1185NdeF and 1185XhoR and inserted into vector pQE30 for protein expression. Point mutations of *rnjB* were introduced by site-directed mutagenesis using QuikChange mutagenesis kit (Agilent Technologies, La Jolla, CA) following the manufacturer's protocol. Wild type or mutant gene fragment containing the *rnjB* gene and its ribosome binding site was PCR amplified and cloned under the control of the nisin promoter of the shuttle vector pMSP3535 for complementation.

*E*. *faecalis rnjA* (*ef2924*) was PCR amplified from the OG1RF genome using primer pair 2924BamF and 2924KpnR.

### Protein overexpression and purification

Recombinant His-tagged *E*. *faecalis* RNase J1, J2 and J2 mutants were overexpressed in *E*. *coli* BL21 and purified using Ni-NTA agarose (Qiagen, Valencia, CA) following the manufacturer’s protocol.

### RNase degradation assays

The exonuclease activities of purified recombinant RNase J1, J2 and the RNase J2 mutants were analyzed by RT-FeDEx assay as described [25]. Briefly, a 30 nt RNA was labeled with a carboxyfluorescein (FAM) group at its 3′-end and hybridized to a 17 nt DNA bearing a 5′-quenching group carboxymethylrhodamine (TAMRA). Degradation of the FAM-labeled RNA substrate releases the fluorophore from the proximity of its quencher and the rate of fluorescence accumulation is measured by a spectrofluorimeter in real time. Kinetic properties of wild-type RNase J1 and J2 were determined using 5 nM for RNase J1 and 200 nM for RNase J2, with substrate RNA concentrations ranging from 0.125 to 2 μM. For comparison of J1, wild-type and mutant J2 exonuclease activities, 5 nM of J1 and 200 nM of each J2 protein with a substrate RNA concentration of 250 μM were used for each assay.

### Surface plasmon resonance (SPR) analysis

Protein-protein interactions of RNase J1/ J2 and J2 mutants were determined by surface plasmon resonance (SPR) on a Biacore T100 as previously described [11]. *E*. *faecalis* RNase J1 was immobilized on a CM5 sensor surface. K_D_ values for these interactions were determined with various concentrations of J2 proteins (400 to 50 nM) run in duplicate and were fitted to the 1:1 binding model using BiaEvaluation software.

### Complementation analysis

pMSP3535 vectors with wild type or *rnjB* derivatives with point mutations were transformed into the *E*. *faecalis rnjB* deletion mutant. Nisin was added to the culture medium at 25ng/ml to induce expression of the *rnjB* gene. The presence of EbpC on the cell surface after nisin induction was determined by flow cytometry as described previously [20].

### Transcriptome analysis

The microarray slides printed with 3,160 primers for *E*. *faecalis* V583 ORFs were used for transcriptome analysis as described previously [26]. Cells grown in BHI to mid-exponential phase were suspended to an OD of 1 and used for RNA extraction. RNAs from three independent cultures for each strain were extracted from using NucleoSpin RNA II kit (Macherey-Nagel, Bethlehem, PA) according to the protocol provided by the supplier. Each RNA preparation was used in two separate dye swap hybridizations (one with parent-Cy3/mutant-Cy5 and the other with parent-Cy5/mutant-Cy3) by labelling with Cy3 or Cy5 during cDNA synthesis from total RNA using Superscript II Kit (Invitrogen) following manufacturer’s instructions. Hybridized microarray slides were scanned (GenePix Pro 5.0; Axon Instruments, Inc.) with independent excitation of the fluorophores Cy3 and Cy5 at 10nm resolution. The signal and background fluorescence intensities were calculated for each DNA spot using the segmentation method of the GENPIXPRO software (Molecular Devices Corp., Union City, CA). For each open reading frame (ORF), ratios of OG1RF to Δ*rnjB* mutant were calculated ratios by averaging ratios for spots in all 6 chips. [27].

### qRT-PCR

Cells grown in BHI to early-log phase, mid-log phase and stationary phase were suspended to an OD of 1 and used for RNA extraction. Total RNA was prepared using NucleoSpin RNAII kit (Macherey-Nagel, Bethlehem, PA) and reverse-transcribed to cDNA using the SuperScript II reverse transcriptase and random primers (Invitrogen, Carlsbad, CA) following the method provided by the manufacturer. Quantitative PCR on cDNA was performed using SYBR green PCR master mix kit and an ABI7900 Real-time PCR system (Invitrogen, Carlsbad, CA). The expression of 23S rRNA and pyrR was analyzed using primer pairs listed in Table 1. For each primer set, a reference curve was established using the genomic DNA purified from wild-type OG1RF cells. The amounts of gene transcripts (ng/ml) obtained for *pyrR* were normalized to the 23S rRNA transcript.

### Extraction of Gls24 and Western blot analysis

Membrane-associated proteins were extracted from *E*. *faecalis* OG1RF and the *rnjB* deletion mutant using Zwittergent as described previously with minor modifications [28]. Briefly, *E*. *faecalis* cells grown to exponential phase were harvested and washed with PBS, and resuspended in 1% of the original culture volume in PBS with 0.2% Zwittergent 3–12 (Calbiochem, La Jolla, Calif.). The suspension was incubated at 25°C for 1 h and dialyzing overnight at 4°C against PBS. Sample was then concentrated by lyophilization, and aliquots stored at -70°C. Equal amounts of total protein from the Zwittergent extracts were loaded on 4%–12% NuPAGE Novex Bis-Tris Gels (Invitrogen, Carlsbad, CA) under reducing conditions in MOPS buffer and transferred to an Immobilon-P PVDF membrane (Millipore, Billerica, MA) according to the manufacturer’s protocol. Membranes were then probed with mouse anti-Gls24 mAb generated in-house against recombinant Gls24 using standard protocols previously described [20], followed by HRP-conjugated goat anti-mouse IgG antibody and developed using TMB substrate system (KPL, Gaithersburg, MD).

### Bile salts resistance assay

The resistance of *E*. *faecalis* OG1RF and its mutants to bile salts was determined using an assay described previously [21]. One O.D. equivalent of overnight cultured cells were harvested and resuspended in 1 mL BHI broth, then diluted 50x in BHI broth with 0.3% bile salts (Fisher, Nazareth, PA)) and incubated at 37°C for 30 min before plating on BHI-agar plates for CFU evaluation. The relative percent survival was determined by comparing the survival rates of mutants (CFU at 30min/CFU at time 0) to that of wild-type OG1RF.

### IV sublethal challenge model

The intravenous challenge experiment was modified from a previously established model [29]. This model is part of a protocol approved by the Animal Welfare Committee, University of Texas Health Science Center at Houston, Houston, TX, USA. In this model, 1x10^8^ bacterial cells of wild-type or *ΔrnjB* strains were injected into mice via the tail vein. After a period of 48 hours, the spleen and kidneys were collected from each mouse, homogenized and serially diluted before plating onto BHI agar plates with rifampicin. Colony growth was enumerated and CFU per gram of organ weight was calculated.

## Results

### *rnjB* is required for normal growth and Ebp pili expression of *Enterococcus faecalis* OG1RF

**Fig 1:**
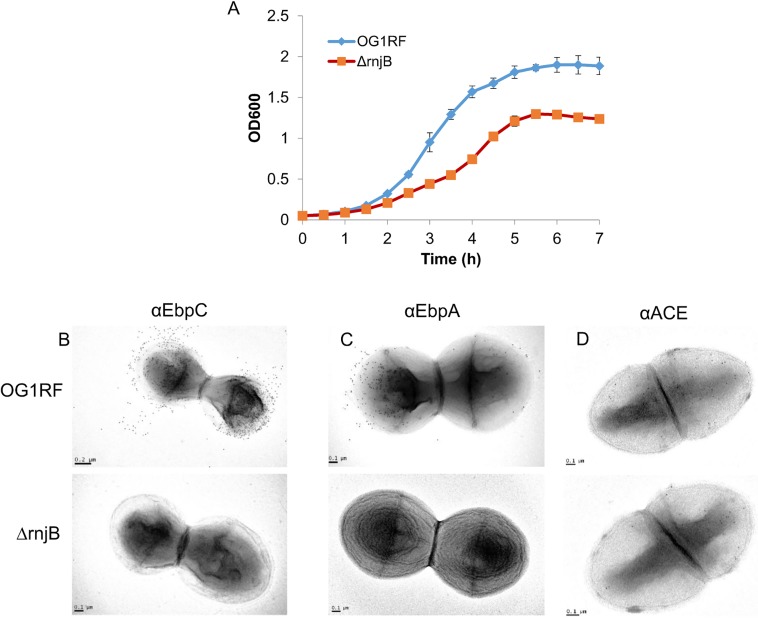
Growth and surface protein expression comparison of OG1RF and *ΔrnjB*. (A) Growth rates of OG1RF and *ΔrnjB* in BHI broth were determined by monitoring OD_600_. Surface expression of EbpC (B), EbpA (C) and Ace (D) on OG1RF and *ΔrnjB* were determined by TEM using anti-EbpC mAb 69, anti-EbpA mAb 9, and anti-Ace mAb 70, respectively.

While deletion of *rnjB* has no known phenotype in *B*. *subtilis*, it is lethal in *Streptococcus pyogenes* [8,30]. In our previous study [20], we constructed an *E*. *faecalis* OG1RF isogenic *rnjB* deletion strain. To determine the effect of the deletion of *rnjB* on *E*. *faecalis* growth, we compared the *in vitro* growth of the wild-type strain OG1RF and *ΔrnjB*. When grown in BHI broth, the *ΔrnjB* mutant had a delayed doubling time to that of wild type and demonstrated a lower saturation density (Fig 1A, 65 min vs. 52 min). However, both wild type and mutant demonstrated all phases of growth, and reached stationary phase around 5 hours. Essentially the same growth profiles were seen in TSBG, another rich medium (data not shown). We conclude that, unlike in other species, deletion of the *E*. *faecalis rnjB* gene is not lethal but does decrease the overall growth rate.

Beyond differences in growth rate or saturation density, the *ΔrnjB* mutant also showed phenotypic differences in surface protein production compared to wild type. We have previously demonstrated that surface expression of EbpC is attenuated in *ΔrnjB* via flow cytometry and Western blot analysis. As shown in Fig 1B using immune-electron microscopy, Ebp pili were broadly distributed on the cell walls distal to the division plane for the wild-type cells. In comparison, EbpC labeling was absent on the *ΔrnjB* mutant, demonstrating that deletion of *rnjB* has a profound effect on Ebp pilus surface display. In EM analysis using an in-house generated anti-EbpA monoclonal antibody (21,22) (Fig 1C), we demonstrated that the surface expression of another Ebp pilin component, EbpA, also could not be detected on the surface of the *rnjB* deletion mutant, further demonstrating that *rnjB* affects production of the Ebp pilin. To study whether *rnjB* specifically affects Ebp pilin or is broadly regulating cell-wall anchored protein display, we examined the surface expression levels in wild-type OG1RF and *ΔrnjB* of another *E*. *faecalis* LPXTG anchored protein, Ace. As shown in Fig 1D, no significant difference was observed in Ace surface expression between OG1RF and *ΔrnjB* as determined by TEM. These results suggest that *rnjB* regulation of surface protein expression is not general to surface anchored proteins, but rather Ebp pilin specific. Unlike *B*. *subtilis*, this clear phenotype of *E*. *faecalis rnjB* may offer complementary opportunities to understand gene function.

### *E*. *faecalis rnjB* encodes an active ribonuclease

**Fig 2:**
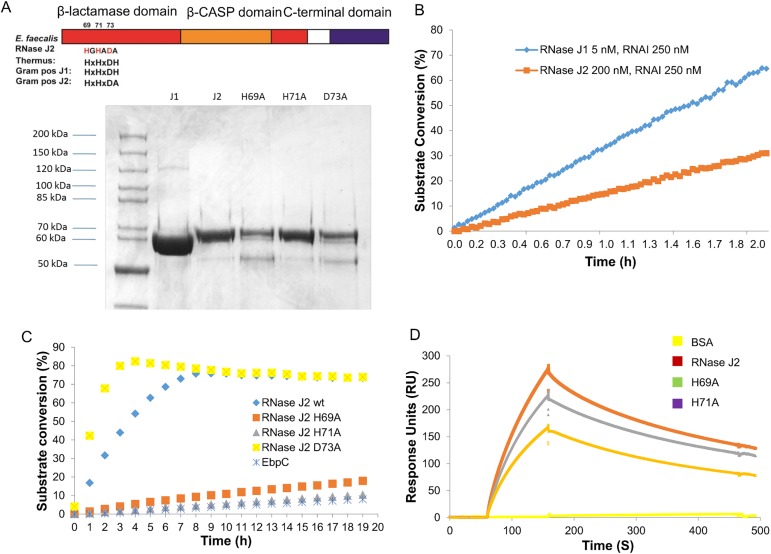
*E*. *faecalis* RNase J1 and J2 are active exonucleases. (A) Diagram depicting the metallo-β-lactamase (red), β-CASP (orange) and dimerization domains. Residues that may be involved in zinc coordination were highlighted. Purified N-His_6_ recombinant *E*. *faecalis* RNase J1, J2 and three point mutations (H69A; H71A; D73A) of J2 were analyzed by SDS-PAGE. (B) and (C) Exonuclease activities of RNase J1, J2 and three J2 mutants were determined by RT-FeDEx analysis. Recombinant EbpC protein served as a negative control for the assay. (D) Binding of *E*. *faecalis* RNase J2 and two mutants (H69A and H71A) (all represented at 100 nM) to immobilized *E*. *faecalis* RNase J1 on a CM5 sensor surface is shown by the SPR sensorgrams.

Sequence analysis revealed that the proteins coded by *E*. *faecalis rnjA* (*ef2924*) and *rnjB* (*ef1185*) are orthologs of the *B*. *subtilis* RNase J1 and J2, respectively. Both *B*. *subtilis* enzymes display endoribonuclease activity, while the exo-ribonuclease activity of J2 is much weaker than that of J1 [10]. In an effort to study *E*. *faecalis* RNase Js’ enzymatic activity, we cloned both *E*. *faecalis rnjA* and *rnjB* genes and expressed recombinant N-terminal His-tagged RNase J1 (M.W. 62.6 KDa) and RNase J2 (M.W. 64.2 KDa) in *E*. *coli* (Fig 2A). The exo-ribonuclease activities of *E*. *faecalis* RNase J1 and J2 were determined by RT-FeDex assay. The kinetic properties of *E*. *faecalis* RNase J1 are comparable to that of *B*. *subtilis* RNase J1 (K_M_ of 0.48 μM and Kcat of 0.167 s^-1^, compared to K_M_ of 0.47 μM and Kcat of 0.58 s^-1^ for the *B*. *subtilis* RNase J1). Due to the high concentration of enzyme required for this assay, we could not accurately measure Kcat and K_M_ for RNase J2. However, RT-FeDex assay clearly demonstrated that RNase J2 is an active enzyme with much lower exonuclease activity as compared to J1(Fig 2B). For example, at a substrate DNA/RNAI concentration of 250 nM, the rate of the *E*. *faecalis* RNase J2 cleavage reaction is about half that of the *E*. *faecalis* J1 reaction, even though the J2 enzyme concentration is 40 times that of J1 (200 nM vs. 5 nM).

Crystal structures of RNase J from *T*. *thermophilus* and *B*. *subtilis* J1 revealed residues that are involved in catalytic zinc ion coordination and RNA recognition [31,32]. In *E*. *faecalis* RNase J2, three of these conserved residues (His 69, His 71 and Asp 73) confirmed by multiple sequence alignments of *E*. *faecalis* RNase J2 with diverse bacteria and archaea were selected for structure-function studies. Amino acid substitution variants of RNase J2 at these three conserved residues (H69A, H71A and D73A) were introduced by site-directed mutagenesis, and the exo-ribonuclease activity of the three point-mutation proteins were determined by RT-FeDEx assay described above. When comparing the exonuclease activities of wild-type and mutant RNase J2s, the wild-type enzyme and one of the mutations (D73A) exhibited enzymatic activities while the other two mutants H69A and H71A were inactive (Fig 2C). SPR analysis of *E*. *faecalis* RNase J1 and J2 interaction (Fig 2D) demonstrated that wild-type RNase J2 and the two inactive mutants (H69A and H71A) bind to *E*. *faecalis* RNase J1 *in vitro*. The K_D_s of these interactions were determined to be 74 nM for the J1-J2 interaction, 81 nM for J1-J2H69A, and 89 nM for J1-J2H71A, all similar to that previously reported in *B*. *subtilis* J1J2 interaction (K_D_ of 80 nM) [32], suggesting that the ability of RNase J1 and J2 to form heteromers is conserved between *Bacillus* and *Enterococcus*. The interactions between *E*. *faecalis* RNase J1 and RNase J2 inactive mutants were comparable to that of the wild-type RNase J2, suggesting that the disruption of RNase J2 active sites does not affect the interaction between J1 and J2.

### *E*. *faecalis* RNase J2 activity is required for Ebp pilus production

**Fig 3:**
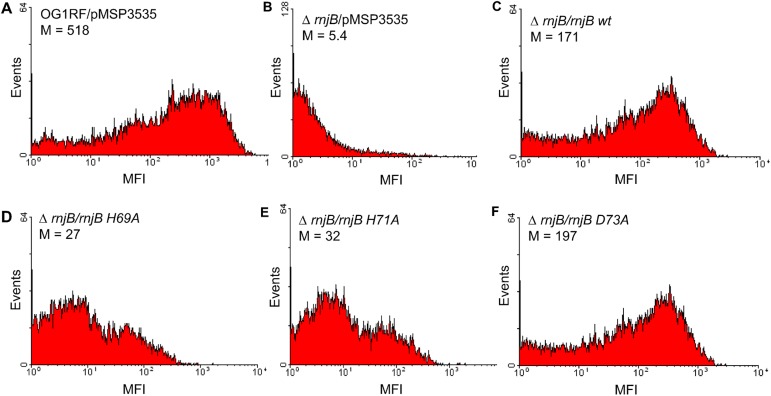
*E*. *faecalis* RNase J2 activity is required for Ebp surface expression. Flow cytometry analysis of (A) OG1RF with empty vector; (B) Δ*rnjB* with empty vector; (C) Δ*rnjB* expressing wild-type RNase J2; (D) Δ*rnjB* expressing inactive RNase J2 with His69 to Ala change; (E) Δ*rnjB* expressing inactive RNase J2 with His71 to Ala change; (F) Δ*rnjB* expressing active RNase J2 with Asp73 to Ala change. Cells grown in BHI medium with 25 ng/ml nisin to stationary phase were labeled with anti-EbpC mAb 69 followed by secondary phycoerythrin anti-mouse IgG conjugate.

We previously demonstrated that *E*. *faecalis rnjB* positively regulates the abundance of *ebp* transcript. To test whether the ribonuclease activity is related to the function of RNase J2 in Ebp pilin gene regulation, we examined production of Ebp pili on the *rnjB* deletion mutant strain complemented with wild-type and mutant RNase J2 genes. As shown in Fig 3, the introduction of the wild-type RNase J2 gene was able to restore pilus expression while mutant genes encoding inactive RNase J2 enzymes failed to do so. This suggests that the ribonuclease activity of RNase J2 is important for its function in pilin regulation.

### Specific transcripts of *E*. *faecalis* affected by absence of RNase J2

To globally characterize the role of RNase J2 in gene expression, we compared the transcriptome of *ΔrnjB* and OG1RF grown in BHI to exponential phase using whole genome DNA microarray analysis. Table 2 lists genes whose transcripts were influenced by deletion of *rnjB*. In summary, compared to OG1RF, mRNA transcripts of 36 genes from the deletion mutant demonstrated a 3-fold decrease with *P* value less than 0.05, or were part of an operon in which at least one member achieved this level of differentiation. In addition, compared to OG1RF, there were 26 mRNA transcripts in the mutant which demonstrated a 2 –fold increase with a *P* value less than 0.05, or were part of a gene cluster in which at least one member achieved this level of differentiation. The dataset of this microarray study was deposited in Gene Expression Omnibus (GEO) with the series accession number GSE95005.

**Table 2:** Genes affected by *rnjB*

Gene ID^a^	Encoded Protein	Change^b^	*P*^*c*^
*ef0077*	conserved hypothetical membrane protein	-4.51	0.006
*ef0078*	conserved hypothetical membrane protein	-4.10	0.002
*ef0079*	gls24 protein	-4.71	0.004
*ef0080*	glsB protein	-5.68	0.012
*ef0081*	conserved hypothetical protein	-5.06	0.013
*ef0082*	transporter, putative	-6.34	0.020
*ef0083*	hypothetical protein	-11.95	0.042
*ef0103*	transcriptional regulator, putative	-3.73	0.030
*ef0104*	arginine deiminase	-3.66	0.020
*ef0105*	ornithine carbamoyltransferase, catabolic	-3.40	0.027
*ef0106*	carbamate kinase	-2.31	0.004
*ef0107*	transcriptional regulator, Crp/Fnr family	-3.18	0.006
*ef0108*	conserved hypothetical protein	-2.94	0.101
*ef0820*	ribosomal protein L25	-8.80	0.034
*ef1090*	EbpR	-2.08	0.018
*ef1091*	EbpA	-7.00	0.121
*ef1092*	EbpB	-12.28	0.035
*ef1093*	EpbC	-12.78	0.102
*ef1094*	SrtC	-4.24	0.026
*ef1096*	conserved hypothetical protein	-6.44	0.072
*ef1097*	Enterococcin V583	-34.64	0.005
*ef1254*	ABC transporter, permease protein	-7.72	0.038
*ef1260*	DNA-binding response regulator	-5.49	0.040
*ef1261*	sensor histidine kinase	-3.97	0.035
*ef1268*	cation-transporting Pase, E1-E2 family	-5.60	0.068
*ef1288*	conserved hypothetical protein	-3.28	0.033
*ef1289*	conserved domain protein	-3.61	0.053
*ef1290*	conserved hypothetical protein	-3.32	0.106
*ef1291*	hypothetical protein	-2.47	0.100
*ef1292*	conserved hypothetical protein	-4.41	0.129
*ef1293*	endolysin	-3.57	0.127
*ef1817*	serine proteinase homolog	-18.68	0.010
*ef1818*	gelatinase	-18.76	0.060
*ef1820*	AgrCfs	-7.60	0.002
*ef1821*	AgrBfs protein	-11.48	0.013
*ef3178*	peptidase, M20/M25/M40 family	-3.08	0.017
*ef0411*	PTS system component	3.70	0.074
*ef0412*	PTS system component	5.56	0.081
*ef0413*	mannitol-1-phosphate 5-dehydrogenase	3.85	0.155
*ef0470*	ribonucleoside-diphosphate reductase 2	2.44	0.106
*ef0471*	ribonucleoside-diphosphate reductase 2	2.94	0.011
*ef0472*	nrdI protein	2.86	0.017
*ef0473*	ribonucleoside-diphosphate reductase 2	2.63	0.002
*ef0635*	amino acid permease	3.13	0.026
*ef0636*	Na+/H+ antiporter	3.13	0.018
*ef1525*	ferric uptake regulator family protein	3.13	0.017
*ef1712*	pyrE orotate phosphoribosyltransferase	5.88	0.019
*ef1713*	pyrF orotidine 5`-phosphate decarboxylase	6.25	0.004
*ef1714*	pyrD-2 dihydroorotate dehydrogenase	8.33	0.030
*ef1715*	pyrDII electron transfer protein, putative	5.88	0.037
*ef1716*	pyrAb carbamoyl-phosphate synthase	10.00	0.043
*ef1717*	pyrAa carbamoyl-phosphate synthase	9.09	0.043
*ef1718*	pyrC dihydroorotase	6.25	0.047
*ef1719*	pyrB aspartate carbamoyltransferase	3.03	0.032
*ef1720*	pyrP permease	3.33	0.131
*ef2160*	transcriptional regulator, MerR family	3.85	0.025
*ef2161*	P-binding protein	2.56	0.013
*ef2162*	tRNA transferase	3.85	0.022
*ef2597*	glycosyl hydrolase, family 1	3.33	0.025
*ef2598*	PTS system component	3.57	0.048
*ef2599*	transcription antiterminator, BglG family	2.56	0.044
*ef2601*	conserved hypothetical protein	2.44	0.013

^a^EF numbers and the encoded protein are from the V583 genome sequenced by TIGR (NCBI ID, NC_004668).

^b^The change represents mRNA expression levels in OG1RF relative to those in the *ΔrnjB* mutant and corresponds to averages of experiments done with three independent cultures. Minus indicates that the expression was lower than in the *ΔrnjB* mutant than in the wild type.

^c^The *P* value from a one-sample *t* test, testing whether the grand mean log_*e*_ ratio was different from 0.0, was significant at the 0.05 or better level for each of the genes tested.

Among the genes that are down-regulated in *ΔrnjB* are the 5 genes in the ebp clusters, (*ebpR*, *A*, *B*, *C* and *srtC*), which is consistent with previous qRT-PCR results [20], and adjacent genes *ef1096* and *ef1097*, the latter encoding a putative antimicrobial peptide [33]. In addition, the virulence related gene cluster, *gls24* operon (ef0077-ef0080), is significantly down regulated in *ΔrnjB* (Table 2).

**Fig 4:**
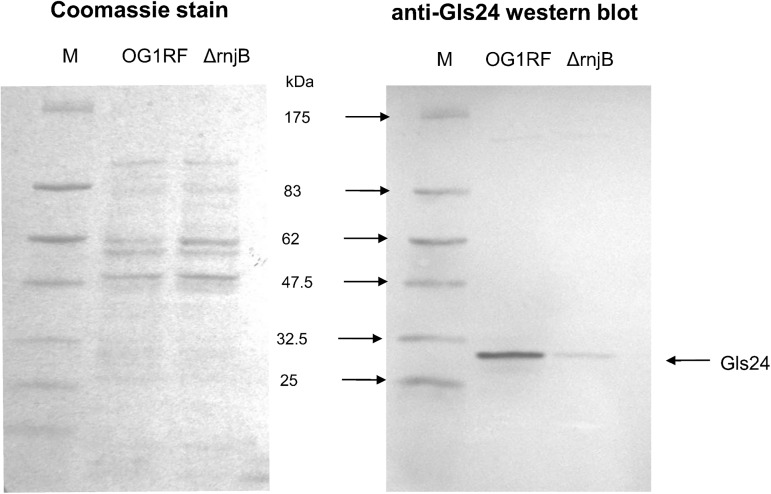
SDS-PAGE and anti-Gls24 Western blot analysis of *E*. *faecalis* Zwittergent surface protein preparation. M: Pre-stained protein marker.

Gls24 (EF0079), first identified as a general stress protein involved in glucose starvation response and bile-salts resistance, also has been shown to play an important role in *E*. *faecalis* virulence, with a *gls24* mutant significantly attenuated in establishing infection in a mouse peritonitis model. [13,21,22,34]. For further exploration of the down-regulation of *gls24* in our transcriptome analysis, we performed Western blot analysis to assess the level of Gls24 expression in an RNase J2 deletion mutant by probing with a Gls24 specific monoclonal antibody we generated using techniques previously described [35,36]. We found that the Gls24 expression level is significantly down regulated in the RNase J2 deletion mutant **(**Fig 4**)**, consistent with observed decreases in transcriptome analysis.

**Fig 5:**
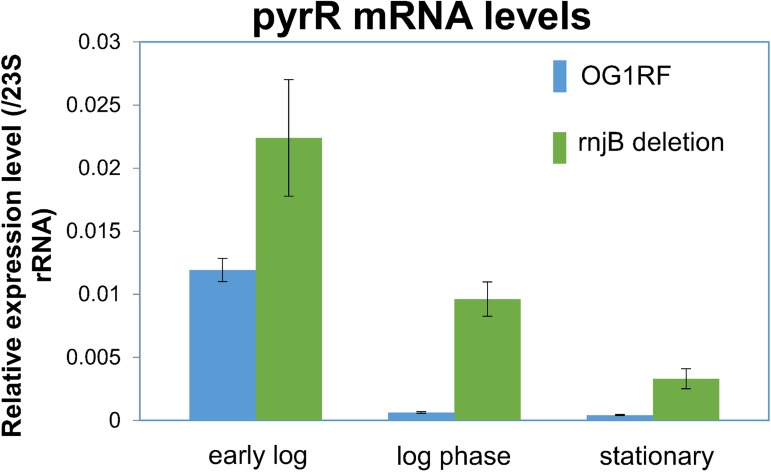
Comparison of transcript level of *pyrR* gene by qRT-PCR. The relative transcript levels of *pyrR* in the rnjB deletion mutant and wild-type OG1RF were determined using 23S rRNA as the internal standard. Experiments were performed in triplicate and error bars represent the standard error of the means.

In contrast to genes down regulated in *ΔrnjB* mentioned above, nine genes of the pyrimidine nucleotide biosynthesis (Pyr) operon, ef1712-1720, *pyrP*,*B*,*Aa*,*Ab*,*C*,*DII*,*D-2*,*F*,*E*, demonstrated 3–8 fold increases in expression levels in *ΔrnjB* compared to wild type. In *Bacillus subtilis* the pyr operon is regulated by the first gene of the operon, pyrR, through an autogenous transcriptional attenuation mechanism [37]. However, the *E*. *faecalis pyrR* (*ef1721*) gene was not represented on the microarray chip, preventing analysis of whether *pyrR* transcript is affected by *rnjB*. To investigate the role of *rnjB* on *pyrR* transcription, we tested the effect of *ΔrnjB* on RNA levels of *pyrR* by qRT-PCR. As shown in Fig 5, mRNA levels of the *pyrR* gene in *ΔrnjB* were 2, 15 and 7.8 -fold higher than those of wild type in early log, mid-log and stationary phase cultures respectively. This result, together with the transcriptome analysis, demonstrated that *rnjB* affects the whole pyr operon, likely through the regulation of pyrR transcription.

### RNase J2 plays important role in *E*. *faecalis* fitness and virulence

**Fig 6:**
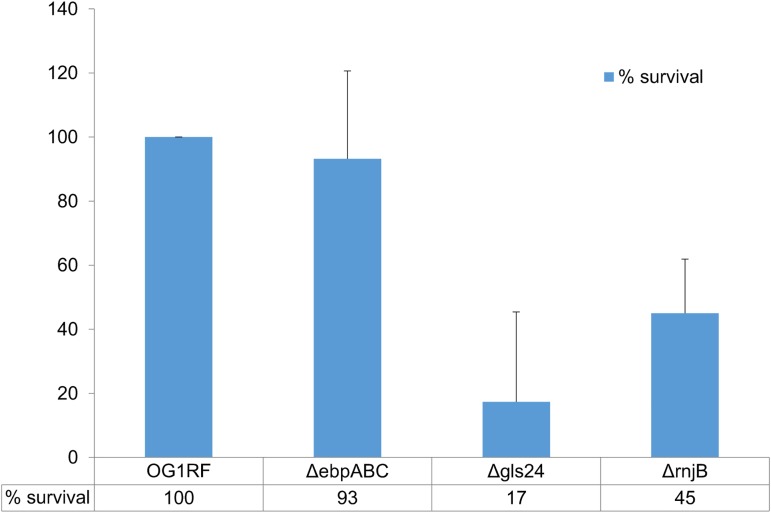
Bile-salt resistance assay. The percent survival of three mutant strains (*ΔebpABC*, *Δgls24 and ΔrnjB*) in BHI medium containing 0.3% bile salt relative to that of wild-type OG1RF was calculated as described in the Materials and Methods section. Each experiment was performed in triplicate and the experiments were repeated 3 times. Error bar represent the standard error of these repeats.

The transcriptome analysis revealed that the *rnjB* gene is required for the expression of multiple virulence-related gene loci, including *ebpABC*, *gls24*, and *ef1097*, a putative antimicrobial peptide [33]. We have previously shown that *ΔrnjB* is attenuated in biofilm formation analyzed by a microplate-binding adherence assay, which correlates with decreased Ebp pili surface display. Gls24, on the other hand, has been reported to be important for *E*. *faecalis* bile salts resistance and virulence [21]. To test whether deletion of *rnjB* has an effect on *E*. *faecalis* stress resistance, a bile-salt resistance assay was performed on *ΔrnjB*, *Δgls24*, and wild-type cells. As demonstrated in Fig 6, the *gls24* deletion strain showed significant reduction in bile-salt resistance. In contrast, the *rnjB* deletion showed modestly reduced resistance to the bile salts compared to wild type (45%) with a P score of 0.03. This result is consistent with the results of transcriptome analysis and Western blot demonstrating that Gls24 expression is decreased but not eliminated in *ΔrnjB*.

**Fig 7:**
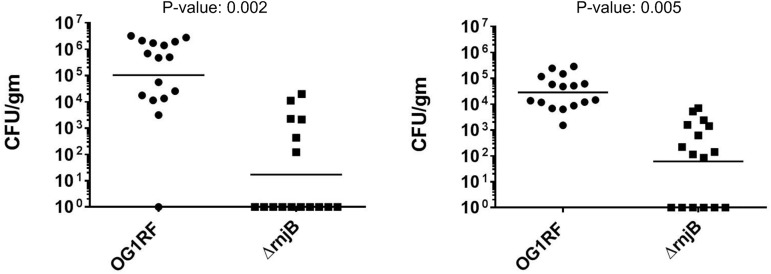
IV infection of ICR mice with *E*. *faecalis* wild type *vs*. *ΔrnjB*. The number of *E*. *faecalis* cells recovered from the kidneys and spleens 48hrs after inoculation are determined as CFU per gram of tissue. On both panels shown on left (Kidney) and right (Spleen), circles and squares represent mice infected with OG1RF (n = 16) and *ΔrnjB* (n = 16), respectively. Horizontal bars represent the geometric mean titers, and the difference of the CFU/gm mean value of wild type and *Δ*rnjB were determined by unpaired *t* tests.

To evaluate the impact of RNase J2 on bacterial virulence *in vivo*, we utilized a sublethal challenge model in mice [29] to study the capacity of wild type vs. *ΔrnjB E*. *faecalis* to establish infection. Two rounds of independent experiments were performed and the combined results are shown in Fig 7. Mice infected with *ΔrnjB* (n = 16) show a 50-fold reduction in mean CFUs in the spleens (*ΔrnjB* 1.2 x10^3^ vs. wild type 6.8x10^4^CFU/gm) and a 400-fold reduction kidneys (*ΔrnjB* 2.2x10^3^ vs. wild type 9.3x10^5^CFU/gm) compared to mice infected with wild type bacteria (n = 16), demonstrating a significant decrease in the ability of the mutant to establish infection (Fig 7). While these results are consistent with the observed inhibition of virulence factor expression in *ΔrnjB* data presented, the described growth delay of the *ΔrnjB* is also a potential factor in overall attenuation. Nonetheless, in totality, these observations are further supportive evidence of *rnjB* playing an important role in Enterococci fitness.

## Discussion

Since discovered over a decade ago [8], many studies have demonstrated an important role for RNase J1 in RNA degradation and maturation. In comparison, the function of RNase J2 has remained largely unknown. Due to lack of an identified substrate and lower enzymatic activity compared to that of RNase J1, it has been speculated that RNase J2 is functionally a duplication of J1 and acts as a substitute or possible regulator of J1[10,11]. However, our study of *E*. *faecalis* RNase J2 suggests that the enzyme may have a very specific role in regulating biological cues for fitness and virulence: First, the ribonuclease activity of RNase J2 is required for Ebp pilus production, suggesting the involvement of RNA degradation or maturation in the process. Second, deletion of RNase J2 in *E*. *faecalis* specifically altered the RNA abundance of 62 genes. Many of these genes are clustered and thus only approximately 20 distinct loci are affected, including several virulence and fitness related genes clusters, indicating specificity of RNase J2 function. Third, among the affected genes are several genes with known roles in gene regulation. For example, PyrR functions as an antiterminator that acts on the leader of the Pyr operon. Thus the effect of RNase J2 on the PyrP, B, Aa, Ab, C, D, F and E genes is likely explained by an effect on PyrR. This role of RNase J2 in antitermination resembles that of *B*. *subtilis* RNase J1 in *thrS* and *trp* operon post-transcription attenuation [38,39]. Similarly, The EbpABC operon is regulated by EbpR and the effect of RNase J2 on EbpABC is likely through EbpR. Therefore, we suspect that RNase J2 directly acts on a small number of mRNAs and/or sRNAs that in turn affect the 62 genes we identified.

The exonuclease activity of *B*. *subtilis* RNase J2 is much lower than RNase J1 [11]. This difference between J1 and J2 activities suggests that J1 and J2 have distinct functions in RNA processing. Crystal structure of *B*. *subtilis* RNase J1 reveals that the Zn^2+^ ion in the J1 catalytic site is coordinated by H74, H76, H142 and D164, while the other Zn^2+^ ion is coordinated by residues D78, H79, D164 and H390 [32]. These residues that coordinate the second Zn^2+^ ion are not conserved in RNase J2s, suggesting a different active site structure. We reported in this manuscript that the exonuclease activity of *E*. *faecalis* RNase J2 is also much lower than its J1 paralog. As shown in Fig 2A, the three residues we chose for amino acid substitution (H69, H71 and D73) are comparable to H74, H76 and D78 in *B*. *subtilis* RNase J1. Mutants of H69 and H71 (equivalent to H74, H76 of the *B*. *subtilis* RNase J1, respectively) significantly reduced *E*. *faecalis* RNase J2 activity, suggesting that these two residues are involved in Zn^2+^ ion coordination. Mutation of D73(equivalent to D78 of the *B*. *subtilis* RNase J1), however, resulted in a mild increase of enzyme activity, suggesting that this residue is not involved in Zn^2+^ ion coordination in J2, further confirming structural differences between RNase J1 and J2. It has been reported that the *B*. *subtilis* RNase J1 and J2 can form a complex with altered enzymatic properties compared to the individual enzymes [11], suggesting a role of RNase J2 as a regulator of J1 through protein-protein interaction. In our study, the inactive mutants of RNase J2 failed to restore Ebp pilus expression, yet the protein-protein interaction with RNase J1 was not affected. This indicates that RNase J2 activity, although much weaker compared with the RNase J1, has unique function in *E*. *faecalis* either through altering RNase J1 activity or independent of RNase J1 activity.

The most interesting observation from the transcriptome analysis is that the *rnjB* deletion mutant showed decreased expression of several known virulence factors including the Ebp proteins and Gls24. Disruption of the *ebp* operon has previously been shown to significantly reduce *E*. *faecalis* biofilm formation and the ability to form vegetation in a rat endocarditis model [22]. Gls24 is an important *E*. *faecalis* stress responder. A *gls24* deletion strain has reduced bile-salt resistance [21]. Additionally, gene disruption experiments have shown that an *E*. *faecalis* mutant lacking a functional *gls24* gene was highly attenuated in a mouse peritoneal lethal challenge model [21] and that disruption decreased virulence in an experimental rat endocarditis model [40]. In our studies of fitness and virulence, we must acknowledge the difficulty in distinguishing attenuation due to a change in pathogenicity factors from those that might be related to the slower growth of *ΔrnjB* mutant seen *in vitro* studies. However, the decrease in virulence in concert with the observed decreased expression of several virulence factors in the RNase J2 deletion mutant, supports a link between RNase J2 and bacterial virulence. This in turn could offer a novel means with which to elucidate regulation mechanisms and design strategies to manipulate virulence factor production. It’s also worthwhile to point out that the Fsr regulon is down-regulated in the *rnjB* deletion mutant by microarray analysis. However, we did not observe the same results in either early-log phase or late phase cultured cells using qRT-PCR analysis. In addition, a gelatin plate assay using overnight culture of OG1RF and *ΔrnjB* showed no difference in end-point gelatinase production (data not shown), further suggesting that the Fsr quorum signal in the *ΔrnjB* mutant is not abolished. Although it has been reported that the *S*. *aureus* Agr system (Fsr analog) plays an important role in RNA regulation, whether *E*. *faecalis* rnjB is involved in Fsr regulation requires further investigation. Altogether, due to the importance of the virulence factors that are affected by *rnjB* in bacterial infection, it can be viewed as promising targets for novel antimicrobial agents. More broadly, the involvement of RNase J2 in bacterial virulence should be further explored in other Gram-positive species, including *S*. *pyogenes* and *S*. *aureus*.

## References

[1] Arraiano CM, Andrade JM, Domingues S, Guinote IB, Malecki M, et al (2010) The critical role of RNA processing and degradation in the control of gene expression. FEMS Microbiol Rev 34: 883–923. doi: 10.1111/j.1574-6976.2010.00242.x 20659169 10.1111/j.1574-6976.2010.00242.x

[2] Condon C, Bechhofer DH (2011) Regulated RNA stability in the Gram positives. Curr Opin Microbiol 14: 148–154. doi: 10.1016/j.mib.2011.01.010 21334965 10.1016/j.mib.2011.01.010 PMC3078962

[3] Daou-Chabo R, Mathy N, Benard L, Condon C (2009) Ribosomes initiating translation of the hbs mRNA protect it from 5'-to-3' exoribonucleolytic degradation by RNase J1. Mol Microbiol 71: 1538–1550. doi: 10.1111/j.1365-2958.2009.06620.x 19210617 10.1111/j.1365-2958.2009.06620.x

[4] Celesnik H, Deana A, Belasco JG (2007) Initiation of RNA decay in Escherichia coli by 5' pyrophosphate removal. Mol Cell 27: 79–90. doi: 10.1016/j.molcel.2007.05.038 17612492 10.1016/j.molcel.2007.05.038 PMC2196405

[5] Deana A, Celesnik H, Belasco JG (2008) The bacterial enzyme RppH triggers messenger RNA degradation by 5' pyrophosphate removal. Nature 451: 355–358. doi: 10.1038/nature06475 18202662 10.1038/nature06475

[6] Condon C (2007) Maturation and degradation of RNA in bacteria. Curr Opin Microbiol 10: 271–278. doi: 10.1016/j.mib.2007.05.008 17560162 10.1016/j.mib.2007.05.008

[7] Mathy N, Benard L, Pellegrini O, Daou R, Wen T, et al (2007) 5'-to-3' exoribonuclease activity in bacteria: role of RNase J1 in rRNA maturation and 5' stability of mRNA. Cell 129: 681–692. doi: 10.1016/j.cell.2007.02.051 17512403 10.1016/j.cell.2007.02.051

[8] Even S, Pellegrini O, Zig L, Labas V, Vinh J, et al (2005) Ribonucleases J1 and J2: two novel endoribonucleases in B.subtilis with functional homology to E.coli RNase E. Nucleic Acids Res 33: 2141–2152. doi: 10.1093/nar/gki505 15831787 10.1093/nar/gki505 PMC1079966

[9] Yao S, Blaustein JB, Bechhofer DH (2007) Processing of Bacillus subtilis small cytoplasmic RNA: evidence for an additional endonuclease cleavage site. Nucleic Acids Res 35: 4464–4473. doi: 10.1093/nar/gkm460 17576666 10.1093/nar/gkm460 PMC1935012

[10] Mader U, Zig L, Kretschmer J, Homuth G, Putzer H (2008) mRNA processing by RNases J1 and J2 affects Bacillus subtilis gene expression on a global scale. Mol Microbiol 70: 183–196. doi: 10.1111/j.1365-2958.2008.06400.x 18713320 10.1111/j.1365-2958.2008.06400.x

[11] Mathy N, Hebert A, Mervelet P, Benard L, Dorleans A, et al (2010) Bacillus subtilis ribonucleases J1 and J2 form a complex with altered enzyme behaviour. Mol Microbiol 75: 489–498. doi: 10.1111/j.1365-2958.2009.07004.x 20025672 10.1111/j.1365-2958.2009.07004.x

[12] Bourgogne A, Singh KV, Fox KA, Pflughoeft KJ, Murray BE, et al (2007) EbpR is important for biofilm formation by activating expression of the endocarditis and biofilm-associated pilus operon (ebpABC) of Enterococcus faecalis OG1RF. J Bacteriol 189: 6490–6493. doi: 10.1128/JB.00594-07 17586623 10.1128/JB.00594-07 PMC1951926

[13] Qin X, Singh KV, Weinstock GM, Murray BE (2000) Effects of Enterococcus faecalis fsr genes on production of gelatinase and a serine protease and virulence. Infect Immun 68: 2579–2586. 10768947 10.1128/iai.68.5.2579-2586.2000 PMC97462

[14] Fox KA, Ramesh A, Stearns JE, Bourgogne A, Reyes-Jara A, et al (2009) Multiple posttranscriptional regulatory mechanisms partner to control ethanolamine utilization in Enterococcus faecalis. PMCID: 2647976. Proc Natl Acad Sci U S A 106: 4435–4440. doi: 10.1073/pnas.0812194106 19246383 10.1073/pnas.0812194106 PMC2647976

[15] DebRoy S, Gebbie M, Ramesh A, Goodson JR, Cruz MR, et al (2014) Riboswitches. A riboswitch-containing sRNA controls gene expression by sequestration of a response regulator. Science 345: 937–940. doi: 10.1126/science.1255091 25146291 10.1126/science.1255091 PMC4356242

[16] Mellin JR, Koutero M, Dar D, Nahori MA, Sorek R, et al (2014) Riboswitches. Sequestration of a two-component response regulator by a riboswitch-regulated noncoding RNA. Science 345: 940–943. doi: 10.1126/science.1255083 25146292 10.1126/science.1255083

[17] Shioya K, Michaux C, Kuenne C, Hain T, Verneuil N, et al (2011) Genome-wide identification of small RNAs in the opportunistic pathogen Enterococcus faecalis V583. PLoS One 6: e23948 doi: 10.1371/journal.pone.0023948 21912655 10.1371/journal.pone.0023948 PMC3166299

[18] Bourgogne A, Garsin DA, Qin X, Singh KV, Sillanpaa J, et al (2008) Large scale variation in Enterococcus faecalis illustrated by the genome analysis of strain OG1RF. Genome Biol 9: R110 doi: 10.1186/gb-2008-9-7-r110 18611278 10.1186/gb-2008-9-7-r110 PMC2530867

[19] Paulsen IT, Banerjei L, Myers GS, Nelson KE, Seshadri R, et al (2003) Role of mobile DNA in the evolution of vancomycin-resistant Enterococcus faecalis. Science 299: 2071–2074. doi: 10.1126/science.1080613 12663927 10.1126/science.1080613

[20] Gao P, Pinkston KL, Nallapareddy SR, van Hoof A, Murray BE, et al (2010) Enterococcus faecalis rnjB is required for pilin gene expression and biofilm formation. J Bacteriol 192: 5489–5498. doi: 10.1128/JB.00725-10 20729365 10.1128/JB.00725-10 PMC2950486

[21] Teng F, Nannini EC, Murray BE (2005) Importance of gls24 in virulence and stress response of Enterococcus faecalis and use of the Gls24 protein as a possible immunotherapy target. J Infect Dis 191: 472–480. doi: 10.1086/427191 15633107 10.1086/427191

[22] Arduin E, Arora S, Bamert PR, Kuiper T, Popp S, et al (2015) Highly reduced binding to high and low affinity mouse Fc gamma receptors by L234A/L235A and N297A Fc mutations engineered into mouse IgG2a. Mol Immunol 63: 456–463. doi: 10.1016/j.molimm.2014.09.017 25451975 10.1016/j.molimm.2014.09.017

[23] Gao P, Pinkston KL, Bourgogne A, Cruz MR, Garsin DA, et al (2013) Library screen identifies Enterococcus faecalis CcpA, the catabolite control protein A, as an effector of Ace, a collagen adhesion protein linked to virulence. J Bacteriol 195: 4761–4768. doi: 10.1128/JB.00706-13 23974022 10.1128/JB.00706-13 PMC3807442

[24] Pinkston KL, Singh KV, Gao P, Wilganowski N, Robinson H, et al (2014) Targeting pili in enterococcal pathogenesis. Infect Immun 82: 1540–1547. doi: 10.1128/IAI.01403-13 24452680 10.1128/IAI.01403-13 PMC3993400

[25] Sinturel F, Pellegrini O, Xiang S, Tong L, Condon C, et al (2009) Real-time fluorescence detection of exoribonucleases. RNA 15: 2057–2062. doi: 10.1261/rna.1670909 19767421 10.1261/rna.1670909 PMC2764478

[26] Aakra A, Vebo H, Snipen L, Hirt H, Aastveit A, et al (2005) Transcriptional response of Enterococcus faecalis V583 to erythromycin. Antimicrob Agents Chemother 49: 2246–2259. doi: 10.1128/AAC.49.6.2246-2259.2005 15917518 10.1128/AAC.49.6.2246-2259.2005 PMC1140525

[27] Bourgogne A, Hilsenbeck SG, Dunny GM, Murray BE (2006) Comparison of OG1RF and an isogenic fsrB deletion mutant by transcriptional analysis: the Fsr system of Enterococcus faecalis is more than the activator of gelatinase and serine protease. J Bacteriol 188: 2875–2884. doi: 10.1128/JB.188.8.2875-2884.2006 16585749 10.1128/JB.188.8.2875-2884.2006 PMC1446981

[28] Xu Y, Jiang L, Murray BE, Weinstock GM (1997) Enterococcus faecalis antigens in human infections. Infect Immun 65: 4207–4215. 9317028 10.1128/iai.65.10.4207-4215.1997 PMC175604

[29] Gentry-Weeks C, Estay M, Loui C, Baker D (2003) Intravenous mouse infection model for studying the pathology of Enterococcus faecalis infections. Infect Immun 71: 1434–1441. doi: 10.1128/IAI.71.3.1434-1441.2003 12595461 10.1128/IAI.71.3.1434-1441.2003 PMC148842

[30] Bugrysheva JV, Scott JR (2010) The ribonucleases J1 and J2 are essential for growth and have independent roles in mRNA decay in Streptococcus pyogenes. Mol Microbiol 75: 731–743. doi: 10.1111/j.1365-2958.2009.07012.x 20025665 10.1111/j.1365-2958.2009.07012.x

[31] Dorleans A, Li de la Sierra-Gallay I, Piton J, Zig L, Gilet L, et al (2011) Molecular basis for the recognition and cleavage of RNA by the bifunctional 5'-3' exo/endoribonuclease RNase J. Structure 19: 1252–1261. doi: 10.1016/j.str.2011.06.018 21893286 10.1016/j.str.2011.06.018

[32] Newman JA, Hewitt L, Rodrigues C, Solovyova A, Harwood CR, et al (2011) Unusual, dual endo- and exonuclease activity in the degradosome explained by crystal structure analysis of RNase J1. Structure 19: 1241–1251. doi: 10.1016/j.str.2011.06.017 21893285 10.1016/j.str.2011.06.017

[33] An Z, Forrest G, Moore R, Cukan M, Haytko P, et al (2009) IgG2m4, an engineered antibody isotype with reduced Fc function. MAbs 1: 572–579. 20073128 10.4161/mabs.1.6.10185 PMC2791314

[34] Singh KV, Qin X, Weinstock GM, Murray BE (1998) Generation and testing of mutants of Enterococcus faecalis in a mouse peritonitis model. J Infect Dis 178: 1416–1420. 9780263 10.1086/314453

[35] Kohler G, Milstein C (1975) Continuous cultures of fused cells secreting antibody of predefined specificity. Nature 256: 495–497. 1172191 10.1038/256495a0

[36] Gao P, Pinkston KL, Nallapareddy SR, van Hoof A, Murray BE, et al (2010) The Enterococcus faecalis rnjB is required for pilin gene expression and biofilm formation. J Bacteriol. 10.1128/JB.00725-10 PMC2950486 20729365

[37] Kinder M, Greenplate AR, Strohl WR, Jordan RE, Brezski RJ (2015) An Fc engineering approach that modulates antibody-dependent cytokine release without altering cell-killing functions. MAbs 7: 494–504. doi: 10.1080/19420862.2015.1022692 25933349 10.1080/19420862.2015.1022692 PMC4622058

[38] Deikus G, Condon C, Bechhofer DH (2008) Role of Bacillus subtilis RNase J1 endonuclease and 5'-exonuclease activities in trp leader RNA turnover. J Biol Chem 283: 17158–17167. doi: 10.1074/jbc.M801461200 18445592 10.1074/jbc.M801461200 PMC2427345

[39] Condon C, Putzer H, Grunberg-Manago M (1996) Processing of the leader mRNA plays a major role in the induction of thrS expression following threonine starvation in Bacillus subtilis. Proc Natl Acad Sci U S A 93: 6992–6997. 8692931 10.1073/pnas.93.14.6992 PMC38922

[40] Nannini EC, Teng F, Singh KV, Murray BE (2005) Decreased virulence of a gls24 mutant of Enterococcus faecalis OG1RF in an experimental endocarditis model. Infect Immun 73: 7772–7774. doi: 10.1128/IAI.73.11.7772-7774.2005 16239583 10.1128/IAI.73.11.7772-7774.2005 PMC1273851

